# High-flow nasal cannula oxygen therapy decreases postextubation neuroventilatory drive and work of breathing in patients with chronic obstructive pulmonary disease

**DOI:** 10.1186/s13054-018-2107-9

**Published:** 2018-08-02

**Authors:** Rosa Di mussi, Savino Spadaro, Tania Stripoli, Carlo Alberto Volta, Paolo Trerotoli, Paola Pierucci, Francesco Staffieri, Francesco Bruno, Luigi Camporota, Salvatore Grasso

**Affiliations:** 1 Dipartimento dell’Emergenza e Trapianti d’Organo (DETO), Sezione di Anestesiologia e Rianimazione, Ospedale Policlinico, Università degli Studi di Bari “Aldo Moro”, Piazza Giulio Cesare 11, Bari, Italy; 2 Dipartimento di Morfologia, Chirurgia e Medicina Sperimentale, Sezione di Anestesiologia e Terapia Intensiva Universitaria, Università degli studi di Ferrara, Ferrara, Italy; 3 Dipartimento di Scienze Biomediche ed Oncologia Umana, Cattedra di Statistica Medica, Università degli Studi Aldo Moro, Bari, Italy; 4 Dipartimento di Medicina Respiratoria e del Sonno, Università degli Studi di Bari “Aldo Moro”, Bari, Italy; 5 Dipartimento dell’Emergenza e Trapianti d’Organo (DETO), Sezione di Chirurgia Veterinaria, Università degli Studi di Bari “Aldo Moro”, Bari, Italy; 6 Department of Adult Critical Care, Guy’s and St Thomas’ NHS Foundation Trust, King’s Health Partners, and Division of Centre of Human Applied Physiological Sciences, King’s College London, London, UK

**Keywords:** High-flow nasal cannula oxygen therapy, Chronic obstructive pulmonary disease, Weaning from mechanical ventilation, Neuroventilatory drive, Work of breathing

## Abstract

**Background:**

The physiological effects of high-flow nasal cannula O_2_ therapy (HFNC) have been evaluated mainly in patients with hypoxemic respiratory failure. In this study, we compared the effects of HFNC and conventional low-flow O_2_ therapy on the neuroventilatory drive and work of breathing postextubation in patients with a background of chronic obstructive pulmonary disease (COPD) who had received mechanical ventilation for hypercapnic respiratory failure.

**Methods:**

This was a single center, unblinded, cross-over study on 14 postextubation COPD patients who were recovering from an episode of acute hypercapnic respiratory failure of various etiologies. After extubation, each patient received two 1-h periods of HFNC (HFNC1 and HFNC2) alternated with 1 h of conventional low-flow O_2_ therapy via a face mask. The inspiratory fraction of oxygen was titrated to achieve an arterial O_2_ saturation target of 88–92%. Gas exchange, breathing pattern, neuroventilatory drive (electrical diaphragmatic activity (EAdi)) and work of breathing (inspiratory trans-diaphragmatic pressure-time product per minute (PTP_DI/min_)) were recorded.

**Results:**

EAdi peak increased from a mean (±SD) of 15.4 ± 6.4 to 23.6 ± 10.5 μV switching from HFNC1 to conventional O_2_, and then returned to 15.2 ± 6.4 μV during HFNC2 (conventional O_2_: *p* < 0.05 versus HFNC1 and HFNC2). Similarly, the PTP_DI/min_ increased from 135 ± 60 to 211 ± 70 cmH_2_O/s/min, and then decreased again during HFNC2 to 132 ± 56 (conventional O_2_: *p* < 0.05 versus HFNC1 and HFNC2).

**Conclusions:**

In patients with COPD, the application of HFNC postextubation significantly decreased the neuroventilatory drive and work of breathing compared with conventional O_2_ therapy.

**Electronic supplementary material:**

The online version of this article (10.1186/s13054-018-2107-9) contains supplementary material, which is available to authorized users.

## Background

High-flow nasal cannula oxygen therapy (HFNC) consists of a totally conditioned, warmed, and humidified air/oxygen blend through a wide-bore nasal cannula at a flow rate between 20 and 60 L/min [1]. Compared with the ‘conventional’ oxygen therapy devices, which deliver gas at 5–20 L/min (conventional O_2_), during HFNC the tracheal inspiratory oxygen fraction (FiO_2_) is more predictable [2] and the mucociliary function is better preserved [3]. In addition, HFNC generates a positive airway pressure (between 2 and 8 cmH_2_O at the pharyngeal level) which resembles positive end-expiratory pressure (PEEP) and is proportional to the administered gas flow rate and varies with the patient breathing pattern (i.e., breathing with the mouth open or closed) [4]. Furthermore, HFNC results in a significant, flow-dependent ‘CO_2_ wash out effect’ of the nasopharyngeal space which decreases the anatomical dead space ventilation and therefore the CO_2_ rebreathing [5]. It seems likely that the overall impact of HFNC on the respiratory function results from the synergistic interaction of the mechanisms described above as well as other, more subtle, and as yet incompletely understood mechanisms [6].

Since its introduction, HFNC has been applied to treat patients with hypoxemic respiratory failure [2, 7–9] and to prevent reintubation in patients at risk of extubation failure [10–12]. In these patients, compared with conventional O_2_ therapy, HFNC improves oxygenation and decreases the work of breathing (WOB) [10, 13]. Studies in patients with stable chronic obstructive pulmonary disease (COPD) in a home-care setting suggest favorable effects on the WOB and gas exchange [14–16]. However, far less known are the physiological effects of HFNC on neuroventilatory drive and WOB in patients with COPD in the critical care setting.

The electric activity of the diaphragm (EAdi) is a ‘processed’ diaphragmatic electromyography signal recorded through an array of electrode pairs mounted on the wall of a nasogastric feeding tube [17]. The EAdi is proportional to the intensity of the electrical stimuli directed to the diaphragm, i.e., the neuroventilatory drive [18–20]. Recently, Bellani and coworkers demonstrated that EAdi can be used to estimate the instantaneous WOB [21].

In this physiological study, we administered HFNC and conventional O_2_ therapy via a face mask postextubation in patients with a background of COPD who had received mechanical ventilation for hypercapnic respiratory failure from various etiologies. The hypothesis of this study was that, in these patients, HFNC decreases the neuroventilatory drive and WOB compared with conventional O_2_ therapy.

## Methods

### Patient selection

We enrolled patients with a background of moderate-severe COPD who were admitted to the intensive care unit (ICU) at the University Hospital of Bari (Italy) between December 2015 and December 2016 and required mechanical ventilation for acute hypercapnic ventilatory failure of various etiologies. The diagnosis of COPD was made by three experts, including one pulmonologist (PP) and two intensivists (SG and TS), and was graded in accordance with the Global Initiative for Chronic Obstructive Lung Disease (GOLD) criteria (http://goldcopd.org/gold-2017-global-strategy-diagnosis-management-prevention-copd/). For each patient, the three assessors reviewed the clinical history, medical records, smoking history, frequency of exacerbation, spirometry data, radiological findings, physical examination, and measurement of static intrinsic PEEP on admission to ICU. The GOLD spirometry criterion for the diagnosis of COPD was a postbronchodilator forced expiratory volume in 1 s/forced vital capacity (FEV_1_/FVC) < 0.7. Based on the FEV_1_ impairment, the severity of COPD was defined as follows: GOLD stage 1 (mild), FEV_1_ ≤ 80% predicted; GOLD stage 2 (moderate), 50% ≤ FEV_1_ < 80% predicted; GOLD stage 3 (severe) 30% ≤ FEV_1_ < 50% predicted; GOLD stage 4 (very severe), FEV_1_ < 30% predicted. The local ethics committee approved the study protocol and informed consent requirements were met according to local regulations (Azienda Ospedaliero-Universitaria Policlinico di Bari Ethic Committee, protocol number: 885/C.E., May 2014).

Patients ready for extubation, as assessed by the treating clinician, were eligible for the study. According to our clinical protocol, the criteria defining readiness for extubation were: a) resolution or improvement of the condition leading to acute respiratory failure; b) set PEEP lower than 6 cmH_2_O and FiO_2_ lower than 0.6 with a PaO_2_/FiO_2_ ratio greater than 150 mmHg; c) arterial pH > 7.35; c) Richmond Agitation Sedation Scale (RASS) between 0 and −1 [22], with no sedation or with a continuous infusion of dexmedetomidine (0.1–1.4 μg/kg/h); and d) ability to trigger the ventilator, i.e., to decrease pressure airway opening (P_AO_) > 3 cmH_2_O during a brief (5–10 s) end-expiratory occlusion test. Other criteria included normothermia and hemodynamic stability unsupported by vasopressors or inotropes, but we allowed low-dose dobutamine (< 5 μg/kg/min) or low-dose dopamine (< 3 μg/kg/min). All patients underwent a 30-min spontaneous breathing trial (SBT) consisting of pressure support ventilation at 5 cmH_2_O with a PEEP of 5 cmH_2_O. Patients were eligible to be included in the study after a successful SBT.

Exclusion criteria were: age < 18 years; < 48 h of invasive mechanical ventilation; presence of a tracheostomy; contraindications to the insertion of the EAdi catheter (e.g., recent upper gastrointestinal surgery, esophageal varices, esophageal trauma); and concomitant neurological or neuromuscular pathologies and/or known phrenic nerve dysfunction. We also excluded patients showing paradoxical abdominal movements or the use of accessory inspiratory muscles. The reason for the latter exclusion criterion is because the correlation between work of breathing and EAdi is valid only if the diaphragm contributes to approximately 75% of the overall WOB [23], and the calculation of WOB from EAdi may be inaccurate if the work carried out by the accessory inspiratory muscles is more than that of the diaphragm.

Before extubation, all patients were ventilated with a Servo-i ventilator (Maquet, Getinge group Critical Care, Solna, Sweden) equipped with the EAdi software (Maquet, Getinge group Critical Care, Solna, Sweden). At the beginning of the study, the standard feeding nasogastric tube was replaced with a 16-Fr, 125-cm EAdi catheter (Maquet, Getinge group Critical Care, Solna, Sweden) unless an EAdi catheter was already in place. The EAdi catheter was first positioned based on the corrected nose-earlobe-xyphoid distance formula, in accordance with the manufacturer’s instructions [24]. Its position was subsequently adjusted using the ventilator EAdi catheter position tool (Servo-i ventilator NAVA software) [24].

### Measurements

Patients were studied in the semirecumbent position. The EAdi signal was collected from the RS232 ventilator port at a sampling rate of 100 Hz (NAVA tracker software, Maquet Getinge group Critical Care, Solna, Sweden) and stored in a personal computer. The NAVA tracker files were subsequently converted and analyzed using the ICULab software package (Kleistek Engineering, Bari, Italy).

The inspiratory EAdi peak (EAdi_PEAK_), the integral of the inspiratory EAdi deflection over time (EAdi_PTP_), the slope of the EAdi from the beginning of inspiration to the peak (EAdi_SLOPE_), the respiratory rate (RR), and the neural inspiratory time (Ti_NEUR_) were measured from the EAdi waveform [25].

Given that all patients were breathing spontaneously, tidal volume (VT) was not measured to avoid any modification in breathing pattern caused by the measurement apparatus.

The pressure generated by the diaphragm (i.e., the trans-diaphragmatic pressure, P_DI_) throughout the inspiratory phase was calculated from the EAdi signal as described by Bellani and coworkers [21]. Briefly, we calculated first the diaphragmatic neuromuscular efficiency (NME), i.e., the ratio between the negative peak in airway opening pressure (P_AO_) during a spontaneous inspiratory effort (recorded during a brief end-expiratory occlusion lasting 5–10 s) and the corresponding peak in the EAdi curve [19, 26, 27]. Since the fall in P_AO_ during a spontaneous inspiratory effort against the occluded airways is, by definition, equal to the corresponding fall in esophageal pressure (P_ES_) [28, 29], the NME is an index of diaphragmatic neuromechanical coupling, and accordingly can be used as a factor to convert the EAdi into P_DI_ (P_DI_ = EAdi × NME) [21]. The inspiratory P_DI_ pressure-time product per breath (PTP_DI/b_) was calculated as the area under the P_DI_ signal. The inspiratory P_DI_ pressure-time product per minute (PTP_DI/min_) was calculated as:

$$ {\mathrm{PTP}}_{\mathrm{DI}/\min }={\mathrm{PTP}}_{\mathrm{DI}/\mathrm{b}}\times \mathrm{RR}. $$

### Study protocol

**Fig. 1:**
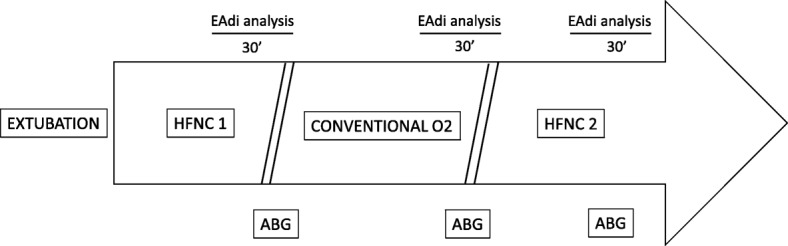
Study protocol timeline. ABG arterial blood gas, Conventional O_2_ period of conventional low flow oxygen therapy through a non-occlusive face mask, EAdi diaphragm electrical activity, HFNC1 first period of high flow nasal cannula oxygen therapy, HFNC2 second period of high flow nasal cannula oxygen therapy

At the beginning of the study, 5–10 min before extubation, the NME was calculated after a brief (5–10 s) end-expiratory occlusion. Immediately after extubation, patients underwent a cross-over protocol with an ON–OFF–ON design, alternating HFNC with conventional O_2_ delivered through a face mask (i.e., HFNC1 – conventional O_2_ therapy – HFNC2), with each phase lasting for 1 h (Fig. 1).

The HFNC was administered through the AIRVO™ 2 system (Fisher & Paykel Healthcare, Auckland, New Zealand) and specific medium/large nasal prongs to fit the size of the nostrils (Fisher & Paykel Healthcare, Auckland, New Zealand). The system allows for the administration of humidified and warmed gas flow (10–60 L/min in the adult configuration). The gas flow was titrated upwards at 5–10 L/min steps starting from 20 L/min, up to the highest flow compatible with patient comfort (maximum allowed flow 60 L/min) [30]. The FiO_2_ was titrated to achieve an hemoglobin oxygen saturation (SaO_2_) target of 88–92%. The temperature of the heated humidifier was set at 37 °C.

In keeping with previous studies [13], the conventional O_2_ therapy was administered through a standard nonocclusive oxygen facial mask connected to a O_2_/air mixer (0–20 L/min). The mask gas flow was set to 10 L/min in all the patients. The FiO_2_ in the mask flow was titrated to achieve the 88–92% SaO_2_ target.

At the end of each study period, arterial blood gas analysis was performed. EAdi and P_DI_ parameters were calculated from the digital recordings of EAdi curve on the last 30 min of each step.

Extubation success was defined as the ability of the patient to breathe spontaneously without signs of respiratory distress and without the requirement of rescue noninvasive ventilation (NIV) for 48 h postextubation. Signs of respiratory distress were defined as: a) paradoxical abdominal movement, use of accessory respiratory muscles, or evidence of respiratory muscle fatigue; b) cardiovascular instability (systolic blood pressure (SBP) > 160 or < 90 mmHg or a 20% change from the pre-SBT values; heart rate (HR) > 120 or < 60 beats/min or 20% change from the pre-SBT values; c) arterial desaturation with SaO_2_ < 88%), hypercapnia, and respiratory acidosis with pH < 7.35; and d) retention of secretions.

### Statistical analysis

The power analysis indicated a sample size of 14 patients with a power of 0.8, a significance level of 0.05 and an expected effect size of 0.25. The effect size refers to the magnitude of variability in an outcome explained by the intervention divided by the total variability of the same outcome measure. We have hypothesized that the variability explained by the study condition in EAdi_PEAK_ had to be at least 5% of total variability that corresponds approximatively to a medium effect size of 0.25. A sphericity correction of 0.8 and a correlation of 0.8 were assumed from pilot measurements. The sample size was determined using the software GPower version 3.1.9.2.

Continuous quantitative variables were summarized as mean ± standard deviation (SD) if normally distributed or as median and interquartile range if non-normally distributed. Comparisons were performed with analysis of variance (ANOVA) for repeated measures or Friedman’s test as appropriate. A *p* value < 0.05 was considered statistically significant, except in the multiple comparison procedure, when the *p* value was adjusted. The analyses were carried out with SAS software v.9.4 for Windows PC.

## Results

**Fig. 2:**
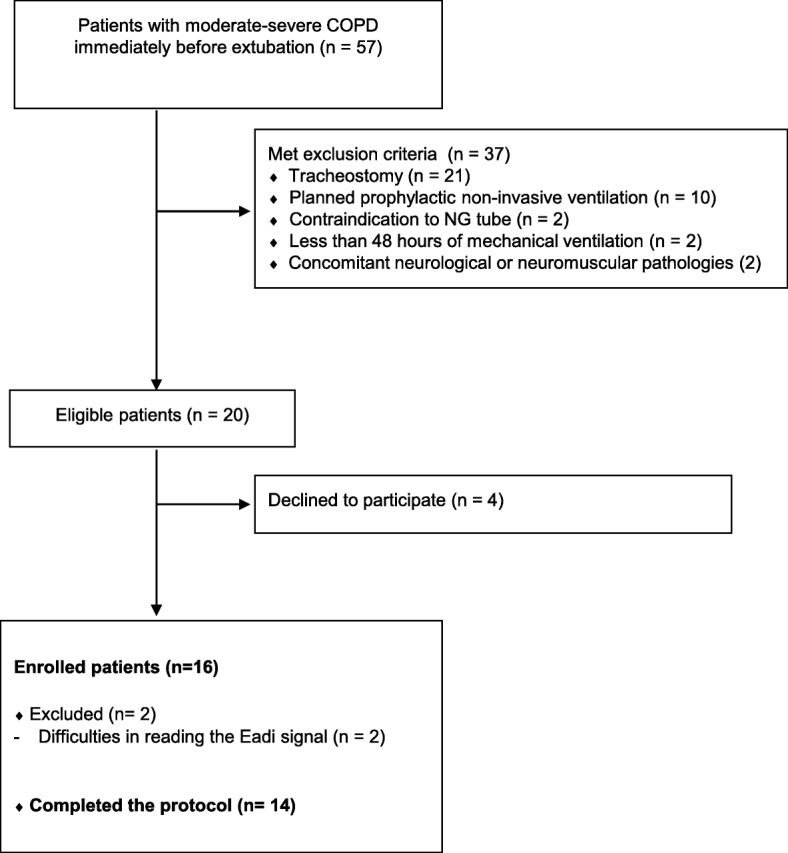
Flow diagram of patient enrollment. COPD chronic obstructive pulmonary disease, EAdi diaphragm electrical activity, NG nasogastric

The CONSORT diagram of our study (Fig. 2) shows that 20 out of the 57 COPD patients admitted to our unit during the study period were eligible for the study and 16 were enrolled. Two patients were excluded from the final analysis because of technical difficulties in recording the EAdi signal. Therefore, the final number of patients was 14. Patient demographics and clinical characteristics are shown in Table 1.

**Table 1:** Main patient characteristics

Patient no.	Gender	Age (years)	SAPS II (at ICU admission)	SOFA (day of study)	Reason for admission to ICU	FEV1/FVC (% predicted)	FEV1 (% predicted)	GOLD stage	Days of MV	Reintubation	ICU length of stay (days)	Hospital outcome
1	M	70	27	3	COPD exacerbation	63	30	3	4	No	7	Survivor
2	M	84	42	4	Postoperative respiratory failure (abdominal sepsis)	68	33	3	14	No	20	Survivor
3	M	70	40	7	Hemorrhagic shock (trauma)	48	53	2	12	No	14	Survivor
4	M	80	36	6	COPD exacerbation	42	55	2	11	Yes	29	Nonsurvivor
5	M	72	58	7	COPD exacerbation	62	55	2	8	No	10	Survivor
6	F	76	58	10	Postoperative respiratory failure (abdominal sepsis)	50	24	4	12	No	13	Survivor
7	M	58	50	10	COPD exacerbation	52	32	3	5	No	11	Survivor
8	M	78	43	5	Postoperative respiratory failure (hip Fracture)	59	27	4	3	Yes	25	Nonsurvivor
9	F	71	54	8	COPD exacerbation	47	25	4	4	No	6	Survivor
10	M	76	31	5	Postoperative respiratory failure (abdominal sepsis)	56	49	3	4	No	5	Survivor
11	M	64	29	3	COPD exacerbation	57	46	3	10	Yes	20	Survivor
12	M	50	10	2	COPD exacerbation	64	64	2	6	No	14	Survivor
13	M	78	35	4	COPD exacerbation	53	59	2	8	Yes	24	Nonsurvivor
14	M	74	42	5	Postoperative respiratory failure (abdominal sepsis)	44	64	2	4	Yes	23	Nonsurvivor
Mean ± SD		71.5 9	39.6 13.2	5.6 2.5					7.5 3.7	Reintubation 5/14 (35.7%)		Survival 10/14 (71.4%)

*COPD* chronic obstructive pulmonary disease, *F* female, *FEV*_*1*_ forced expiratory volume in 1 s, *FVC* forced vital capacity, *GOLD* Global Initiative for Chronic Lung Disease, *ICU* intensive care unit, *M* male, *MV* mechanical ventilation, *SAPS* Simplified Acute Physiology Score, *SOFA* Sequential Organ Failure Assessment

Five patients (31.5%) failed the initial extubation attempt and were reintubated. Patients who required reintubation were similar to the ones who were successfully extubated in terms of age, reason for ICU admission (COPD exacerbation vs other causes), days of mechanical ventilation, COPD severity (based on FEV_1_, FEV_1_/FVC ratio, GOLD stage, Simplified Acute Physiology Score (SAPS) II on admission, and Sequential Organ Failure Assessment (SOFA) score; Additional file 1).

Four patients (25%) died after a mean (±SD) ICU length of stay of 25.2 ± 2.6 days. The cause of death for two patients was septic shock and multiple organ failure, while the other two died of right cardiac failure and cardiogenic shock.

### Breathing pattern and gas exchange

Table 2 shows the breathing pattern and gas exchange recorded for each of the three experimental conditions. To achieve the oxygenation target (SaO_2_ between 88 and 92%), the applied FiO_2_ during HFNC1 and HFNC2 periods was 0.46 ± 0.1 and 0.46 ± 0.12, respectively (*p* = not significant), whereas during the conventional (mask) O_2_ period it was 0.80 ± 0.19. However, a comparison between the FiO_2_ during HFNC and conventional O_2_ is not meaningful given the difference in the delivered gas flow rates. Indeed, the FiO_2_ delivered during conventional O_2_ therapy is almost certainly overestimated since the patient’s inspiratory flow is higher than the mask flow (10 L/min) and, therefore, the difference between patient inspiratory flow and the mask flow is provided by room air_._ This also makes any comparison between the PaO_2_/FiO_2_ in the different experimental conditions inappropriate. Respiratory rate, Ti_NEUR_, arterial PCO_2_, and pH remained similar throughout the study (Table 2).

**Table 2:** Breathing pattern and gas exchange in different experimental conditions

	HFNC1	Conventional O_2_	HFNC2
RR (breaths/min)	20.5 ± 2.9	21.4 ± 4	20.0 ± 1.9
Ti_NEUR_ (s)	0.92 ± 0.21	0.95 ± 0.22	0.92 ± 0.17
pH	7.45 ± 0.07	7.44 ± 0.08	7.46 ± 0.08
PaCO_2_ (mmHg)	49.9 ± 11.9	51.8 ± 12.7	50.1 ± 12.6
HCO_3_^−^ (mEq/L)	30.9 ± 7.6	31.3 ± 7.8	31.4 ± 8.4
PaO_2_ (mmHg)	75.1 ± 6.9	72.9 ± 8.6	81.2 ± 8
Applied FiO_2_^a^	0.46 ± 10	0.80 ± 0.19^b,c^	0.46 ± 0.12

Data are expressed as mean ± standard deviation

*Conventional O*_*2*_ conventional low flow oxygen therapy through a nonocclusive face mask, *FiO*_*2*_ inspiratory oxygen fraction, *HFNC* high-flow nasal cannula oxygen therapy, *PaCO*_*2*_ arterial partial carbon dioxide pressure, *PaO*_*2*_ arterial partial oxygen pressure, *RR* respiratory rate, *Ti*_*NEUR*_ neural inspiratory time

^a^ The FiO_2_ delivered during conventional O_2_ therapy is overestimated since the patient’s inspiratory flow was higher than the mask flow (10 L/min) and, therefore, the difference between patient inspiratory flow and mask flow was taken by room air; this makes inappropriate any comparison between the PaO_2_/FiO_2_ ratio in the different experimental conditions

^b^ Different from HFNC1, ANOVA, with Bonferroni correction

^c^ Different from HFNC2, ANOVA, with Bonferroni correction

### Neuroventilatory drive and work of breathing

**Fig. 3:**
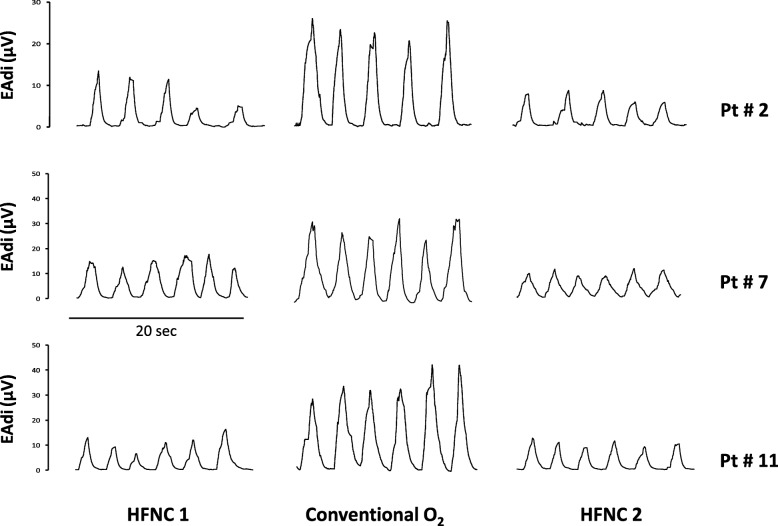
Experimental record showing the diaphragm electrical activity (EAdi) in the three experimental conditions in three representative patients. Conventional O_2_ period of conventional low flow oxygen therapy through a non-occlusive face mask, HFNC1 first period of high flow nasal cannula oxygen therapy, HFNC2 second period of high flow nasal cannula oxygen therapy

**Fig. 4:**
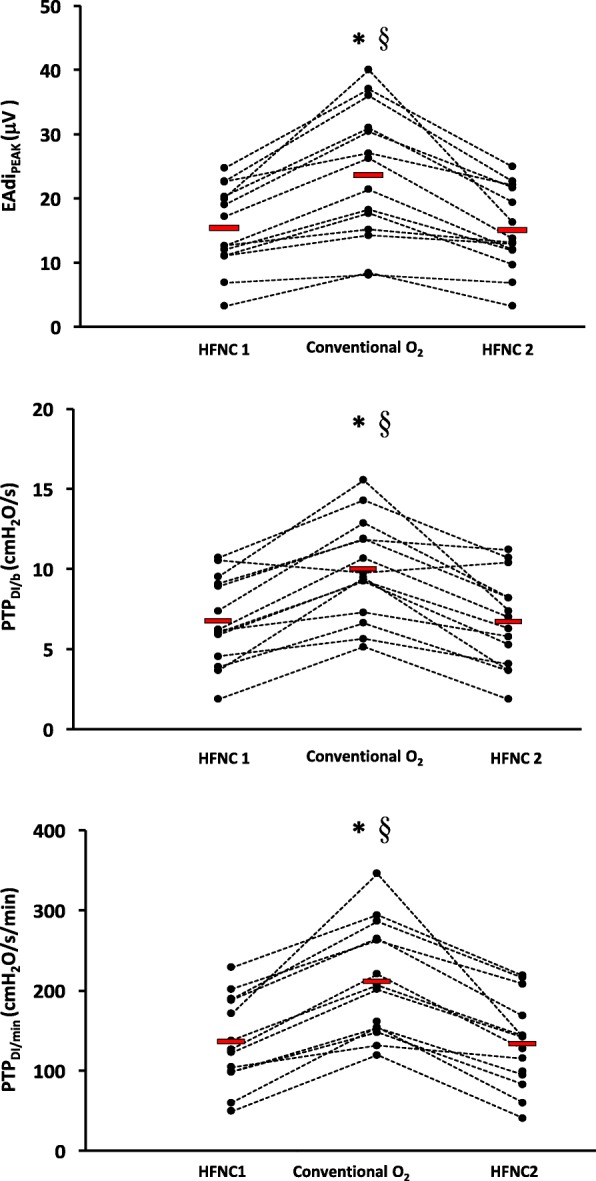
Trend of the neuroventilatory drive, as expressed by the diaphragm electrical activity peak EAdi_PEAK_, and of work of breathing, as expressed by the inspiratory P_DI_ pressure-time product per breath (PTP_DI/b)_ and per minute (PTP_DI/min_). *Significant difference compared to the HFNC1 period (ANOVA with Bonferroni correction); ^§^Significant difference compared to the HFNC2 period (ANOVA with Bonferroni correction). Conventional O_2_ period of conventional low flow oxygen therapy through a non-occlusive face mask, HFNC1 first period of high flow nasal cannula oxygen therapy, HFNC2 second period of high flow nasal cannula oxygen therapy

Figure 3 shows EAdi traces obtained at the end of each study period in three representative patients. In all patients, the neuroventilatory drive, expressed by the EAdi waveforms, clearly increased between HFNC1 and conventional O_2_ therapy and decreased again when the HFNC was reinstituted (HFNC2). Table 3 shows that neuroventilatory drive (EAdi_PEAK_) and work of breathing (PTP_DI/b_ and PTP_DI/min_) increased significantly while on conventional O_2_ therapy, and decreased again when HFNC was reinstituted. Figure 4 shows the individual changes in these parameters during the three study periods.

**Table 3:** Neuroventilatory drive and work of breathing parameters

	HFNC1	Conventional O_2_	HFNC2
EAdi_PEAK_ (μV)	15.4 ± 6.4	23.6 ± 10.5^a,b^	15.2 ± 6.4
EAdi_PTP_ (μV/s)	13.7 ± 6.5	21.1 ± 11.8^a,b^	12.1 ± 5.2
EAdi_SLOPE_	18.6 ± 6.5	24 ± 14.7^a,b^	17.6 ± 10.2
PTP_DI/b_ (cmH_2_O/s)	6.7 ± 2.7	9.9 ± 3.1^a,b^	6.7 ± 2.8
PTP_DI/min_ (cmH_2_O/s/min)	135 ± 60	211 ± 70^a,b^	132 ± 56

Data are expressed as mean ± standard deviation

*Conventional O*_*2*_ conventional low flow oxygen therapy through a nonocclusive face mask, *EAdi*_*PEAK*_ diaphragm electrical activity peak, *EAdi*_*PTP*_ EAdi deflection inspiratory area, *EAdi*_*SLOPE*_ EAdi slope from the beginning of inspiration to EAdi_PEAK_, *HFNC* high-flow nasal cannula oxygen therapy, *PTP*_*DI/b*_ inspiratory trans-diaphragmatic pressure-time product per breath, *PTP*_*DI/min*_ inspiratory trans-diaphragmatic pressure-time product per minute

^a^ Different from HFNC1, ANOVA, with Bonferroni correction

^b^ Different from HFNC2, ANOVA, with Bonferroni correction

We were not able to find any significant differences between patients that were reintubated and patients successfully extubated in terms of EAdi parameters or work of breathing during each experimental condition (Additional file 2). For the same parameters, there were no significant differences between patients admitted for hypercapnic respiratory failure due to an exacerbation of COPD and patients with a background of COPD but whose hypercapnic respiratory failure was due to other precipitating causes (Additional file 3).

## Discussion

This study shows that postextubation HFNC significantly decreases the neuroventilatory drive and work of breathing in patients with COPD who had received mechanical ventilation for hypercapnic respiratory failure due to various etiologies.

The EAdi reflects the rate of discharge of the phrenic nerve and therefore it is a measure of the neuroventilatory drive [17–20, 31, 32]. Thus, our data clearly show that HFNC decreases the neuroventilatory drive (EAdi_PEAK_ and EAdi_SLOPE_) compared with conventional O_2_ therapy. Neuroventilatory drive and work of breathing are key factors for the weaning process and an excessive respiratory drive predicts weaning failure [26, 32]. In fact, a high ventilatory drive may be associated either with excessive mechanical load posed on the inspiratory muscles, diaphragm weakness, or inappropriately high activation of the respiratory centers due to pain, fever, anxiety, and acidosis [32]. In a mixed population of critically ill patients, Liu and coworkers found that an EAdi_PEAK_ lower than 15–20 μV during a spontaneous breathing trial (T-tube) was associated with weaning success [26]. Similar results were recently obtained in two other studies by Dres et al. [33] and Barwing et al. [34]. In our study, we found that the EAdiP_EAK_ was below this threshold in most of the patients during both HFNC periods (Fig. 4), while it was on average 1.5-times higher than this threshold during conventional O_2_. Accordingly, considering that COPD patients are intrinsically at risk of weaning failure [35], our results are potentially clinically relevant.

Although the work of breathing is proportional to the neuroventilatory drive, its absolute value depends on the ability of the respiratory muscles to convert the electrical stimuli into mechanical contraction (electromechanical coupling) [18, 32]. We measured the work of breathing in terms of PTP_DI_ per breath and per minute, a well-known index of respiratory muscle oxygen consumption (Table 3 and Fig. 4). According to physiological studies in mixed populations of critically ill patients, an ‘acceptable’ PTP_DI/min_ is between 50 and 150 cmH_2_O/s/min [36, 37]. The PTP_DI/min_ was in this range in 64.3% of our patients (i.e., 9/14) both during HFNC1 and HFNC2 periods, whereas the PTP_DI/min_ was above this acceptable range in 78.6% of patients during the conventional O_2_ period (i.e., 11/14) (Fig. 3).

According to the 2017 European Respiratory Society–American Thoracic Society (ERS/ATS) guidelines [38], COPD patients benefit from noninvasive ventilation to prevent reintubation. Therefore, it would have been of interest to compare the physiological effects of HFNC and NIV in our patients. However, at the time of the study, postextubation preventative NIV was not applied on a routine basis in our institution. Interestingly, a recent study by Hernandez et al. showed that HFNC is noninferior to NIV in preventing acute postextubation respiratory failure in patients at “high risk” of postextubation respiratory failure, including patients older than 65 years or those with heart failure, moderate to severe COPD, an Acute Physiology and Chronic Health Evaluation (APACHE) II score higher than 12 on extubation day, a body mass index of more than 30, those with airway patency problems, and, finally, patients with difficult or prolonged weaning [11].

Further studies are needed to assess the beneficial mechanisms of HFNC in COPD patients. We speculate that two mechanisms are of particular relevance: a) the HFNC “PEEP” effect [14], that may have counterbalanced the flow-limited intrinsic positive end-expiratory pressure (PEEPi), and b) the “CO_2_ wash-out” effect of the anatomical dead space [5] that may have decreased the diaphragmatic workload. The better preservation of the mucociliary function as compared with conventional O_2_ therapy may have been an adjunctive mechanism [3], but we believe that it was less important since the cross-over periods were relatively short.

In hypoxemic patients, Mauri et al. [39] and Maggiore et al. [10] found that HFNC significantly decreased RR compared with conventional O_2_ therapy. Mauri estimated the VT through electrical impedance tomography (EIT) and found that it remained stable. In contrast, in our COPD patients, the RR remained unchanged (Table 2), while we have no data on VT since patients were breathing spontaneously and we wanted to avoid any modification in breathing pattern caused by the measurement apparatus. However, the VT likely increased since animal studies show that VT is proportional to the electrical activity of the diaphragm during unassisted spontaneous breathing [40]. Based on this hypothesis, in our patients, the response to HFNC removal during the conventional O_2_ period would have been similar to the physiological response to a sudden increase in respiratory workload during to CO_2_ rebreathing, i.e., to maintain the RR as constant and to increase the VT [41, 42]. The different impact of HFNC on RR between our study and those of Mauri and Maggiore could be explained by the different background of the respiratory failure of the studied patients (hypoxemic versus hypercapnic).

In our study, similar to previous studies [13], we used a standard, nonocclusive oxygen facial mask with a fixed gas flow of 10 L/min in all the patients during the conventional O_2_ study step (see the Methods section). Hence, in our patients the peak inspiratory flow was very likely greater than the mask gas flow and therefore the true fraction of inhaled oxygen was lower than the one provided by the mask. The “Venturi Mask” is a high-flow oxygen delivery system that provides 35–45 L/min of a mixture of oxygen and air with a delivered FiO_2_ of 0.24–0.6 by taking advantage of the Bernoulli principle [43]. By using a Venturi Mask instead of the standard mask, it is possible that we would have better matched the patient’s inspiratory flow during the conventional O_2_ study period. One could also speculate that a higher mask flow could have other effects in terms of CO_2_ washout from the mask or from the airways, but we are not aware of studies comparing Venturi mask and HFNC.

We must acknowledge some study limitations. First, we studied a population of patients with COPD that was admitted to the ICU with hypercapnic ventilatory failure due to various etiologies (Table 1). Only 8/14 (57%) of COPD patients were admitted because of a COPD exacerbation, while the other 6 (43%) received mechanical ventilation for postoperative ventilatory failure. In this regard, our population could be deemed as heterogenous. However, we point out that: 1) our study was conducted in the postextubation phase when the primary reason for the acute respiratory failure had resolved or at least improved (see Methods); and 2) all our patients had moderate to very severe COPD according to the GOLD classification. Second, we were not able to measure several respiratory parameters during spontaneous breathing (VT, PEEPi, inspiratory flow) that could have provided us with a more complete interpretation of the treatment effect. However, our study was conducted in spontaneously breathing patients and we sought to avoid any modification in breathing pattern caused by the measurement apparatus. Third, we measured the work of breathing based on a method recently validated by Bellani and coworkers [21], but the correlation between work of breathing and EAdi may be misleading if the contraction of the accessory inspiratory muscles is dominant compared with the diaphragmatic contraction. Indeed, the estimation of work of breathing from EAdi assumes that the diaphragm contributes approximately 75% to the overall WOB (as occurs in normal conditions) [23]. However, we assessed all patients for signs of paradoxical abdominal motion and use of accessory inspiratory muscles throughout the study. In addition, the method described by Bellani et al. assumes a linear relationship between EAdi and P_DI_ at different lung volumes based on a close correlation at different lung volumes between the P_DI_ obtained from the esophageal pressure and the P_DI_ obtained through the formula EAdi × NME [21]. However, Bellani et al. studied patients ventilated with different levels of pressure support ventilation (PSV) and neurally adjusted ventilatory assist (NAVA) while we studied spontaneously breathing patients. Of note, other authors showed a nonlinearity between diaphragmatic efficiency and lung volumes, but only for intense diaphragmatic contractions [19]. Fourth, we studied a small patient number that, while appropriate for a physiologically oriented study, weakens any speculation on the clinical outcomes (e.g., ICU and hospital length of stay and reintubation rate).

## Conclusions

In conclusion, we found that HFNC, as compared with conventional O_2_ therapy, significantly decreases the neuroventilatory drive and the work of breathing in patients with COPD recovering from an episode of acute respiratory failure after a planned extubation.

## Additional files

Additional file 1: Independent sample *t* tests. Comparison between patients who required reintubation and patients who were successfully extubated in terms of age, reason for ICU admission (COPD exacerbation versus other causes), days of mechanical ventilation, COPD severity (based on FEV_1_, FEV_1_/FVC ratio, GOLD stage, SAPS II on admission, and SOFA score). (DOCX 26 kb)

Additional file 2: Independent sample *t* tests. Comparison between patients who required reintubation and patients who were successfully extubated in terms of EAdi parameters or work of breathing during each experimental condition. (DOCX 42 kb)

Additional file 3: Independent sample *t* tests. Comparison between patients admitted for hypercapnic respiratory failure due to an exacerbation of COPD and patients with a background of COPD but whose hypercapnic respiratory failure was due to other precipitating causes in terms of EAdi parameters or work of breathing during each experimental condition. (DOCX 60 kb)

## Abbreviations

COPD: Chronic obstructive pulmonary disease
EAdi: Electric activity of the diaphragm
FEV_1_: Forced expiratory volume in 1 s
FiO_2_: Inspiratory oxygen fraction
FVC: Forced vital capacity
GOLD: Global Initiative for Chronic Lung Disease
HFNC: High-flow nasal cannula oxygen therapy
ICU: Intensive care unit
NIV: Noninvasive ventilation
NME: Neuromuscular efficiency
P_AO_: Pressure airway opening
PEEP: Positive end-expiratory pressure
PEEPi: Intrinsic positive end-expiratory pressure
PTP_DI/b_: Pressure-time product per breath
PTP_DI/min_: Pressure-time product per minute
RR: Respiratory rate
SaO_2_: Arterial hemoglobin oxygen saturation
SBP: Systolic blood pressure
SBT: Spontaneous breathing trial
Ti_NEUR_: Neural inspiratory time
VT: Tidal volume
WOB: Work of breathing
