# Supplementary material for: High-flow nasal cannula oxygen therapy decreases postextubation neuroventilatory drive and work of breathing in patients with chronic obstructive pulmonary disease
Source: Crit Care. 2018 Aug 2;22:180. doi: 10.1186/s13054-018-2107-9 (PMC6091018; doi:10.1186/s13054-018-2107-9)
Supplement: Supplementary file 1 — Independent sample t tests. Comparison between patients who required reintubation and patients who were successfully extubated in terms of age, reason for ICU admission (COPD exacerbation versus other causes), days of mechanical ventilation, COPD severity (based on FEV1, FEV1/FVC ratio, GOLD stage, SAPS II on admission, and SOFA score). (DOCX 26 kb) [file 13054_2018_2107_MOESM1_ESM.docx]

Independent samples t-test

| Sample 1 | |
| --- | --- |
| Variable | Age |
| Filter | No Reintubation |
| Sample 2 | |
| Variable | Age |
| Filter | Reintubation |

|  | Sample 1 | Sample 2 |
| --- | --- | --- |
| Sample size | 9 | 5 |
| Arithmetic mean | 69.6667 | 74.8000 |
| 95% CI for the mean | 61.9035 to 77.4298 | 66.8301 to 82.7699 |
| Variance | 102.0000 | 41.2000 |
| Standard deviation | 10.0995 | 6.4187 |
| Standard error of the mean | 3.3665 | 2.8705 |

| F-test for equal variances | P = 0.398 |
| --- | --- |

## T-test (assuming equal variances)

| Difference | 5.1333 |
| --- | --- |
| Standard Error | 5.0426 |
| 95% CI of difference | -5.8536 to 16.1203 |
| Test statistic t | 1.018 |
| Degrees of Freedom (DF) | 12 |
| Two-tailed probability | P = 0.3288 |

Independent samples t-test

| Sample 1 | |
| --- | --- |
| Variable | Re_intubation |
| Filter | Reason_of_admission_in_ICU=0 |
| Sample 2 | |
| Variable | Re_intubation |
| Filter | Reason_of_admission_in_ICU=1 |

|  | Sample 1 | Sample 2 |
| --- | --- | --- |
| Sample size | 8 | 6 |
| Arithmetic mean | 0.3750 | 0.3333 |
| 95% CI for the mean | -0.05768 to 0.8077 | -0.2086 to 0.8753 |
| Variance | 0.2679 | 0.2667 |
| Standard deviation | 0.5175 | 0.5164 |
| Standard error of the mean | 0.1830 | 0.2108 |

| F-test for equal variances | P = 0.967 |
| --- | --- |

## T-test (assuming equal variances)

| Difference | -0.04167 |
| --- | --- |
| Standard Error | 0.2792 |
| 95% CI of difference | -0.6501 to 0.5668 |
| Test statistic t | -0.149 |
| Degrees of Freedom (DF) | 12 |
| Two-tailed probability | P = 0.8839 |

Independent samples t-test

| Sample 1 | |
| --- | --- |
| Variable | Days_of_MV |
| Filter | No Reintubation |
| Sample 2 | |
| Variable | Days_of_MV |
| Filter | Reintubation |

|  | Sample 1 | Sample 2 |
| --- | --- | --- |
| Sample size | 9 | 5 |
| Arithmetic mean | 7.6667 | 7.2000 |
| 95% CI for the mean | 4.5920 to 10.7413 | 2.7751 to 11.6249 |
| Variance | 16.0000 | 12.7000 |
| Standard deviation | 4.0000 | 3.5637 |
| Standard error of the mean | 1.3333 | 1.5937 |

| F-test for equal variances | P = 0.878 |
| --- | --- |

## T-test (assuming equal variances)

| Difference | -0.4667 |
| --- | --- |
| Standard Error | 2.1530 |
| 95% CI of difference | -5.1577 to 4.2244 |
| Test statistic t | -0.217 |
| Degrees of Freedom (DF) | 12 |
| Two-tailed probability | P = 0.8320 |

Independent samples t-test

| Sample 1 | |
| --- | --- |
| Variable | FEV1 |
| Filter | No Reintubation |
| Sample 2 | |
| Variable | FEV1 |
| Filter | Reintubation |

|  | Sample 1 | Sample 2 |
| --- | --- | --- |
| Sample size | 9 | 5 |
| Arithmetic mean | 40.5556 | 50.2000 |
| 95% CI for the mean | 29.2121 to 51.8990 | 32.1339 to 68.2661 |
| Variance | 217.7778 | 211.7000 |
| Standard deviation | 14.7573 | 14.5499 |
| Standard error of the mean | 4.9191 | 6.5069 |

| F-test for equal variances | P = 0.947 |
| --- | --- |

## T-test (assuming equal variances)

| Difference | 9.6444 |
| --- | --- |
| Standard Error | 8.1929 |
| 95% CI of difference | -8.2062 to 27.4951 |
| Test statistic t | 1.177 |
| Degrees of Freedom (DF) | 12 |
| Two-tailed probability | P = 0.2619 |

Independent samples t-test

| Sample 1 | |
| --- | --- |
| Variable | FEV1_FVC |
| Filter | No Reintubation |
| Sample 2 | |
| Variable | FEV1_FVC |
| Filter | Reintubation |

|  | Sample 1 | Sample 2 |
| --- | --- | --- |
| Sample size | 9 | 5 |
| Arithmetic mean | 56.6667 | 51.0000 |
| 95% CI for the mean | 50.6755 to 62.6578 | 41.5031 to 60.4969 |
| Variance | 60.7500 | 58.5000 |
| Standard deviation | 7.7942 | 7.6485 |
| Standard error of the mean | 2.5981 | 3.4205 |

| F-test for equal variances | P = 0.955 |
| --- | --- |

## T-test (assuming equal variances)

| Difference | -5.6667 |
| --- | --- |
| Standard Error | 4.3205 |
| 95% CI of difference | -15.0802 to 3.7469 |
| Test statistic t | -1.312 |
| Degrees of Freedom (DF) | 12 |
| Two-tailed probability | P = 0.2142 |

Independent samples t-test

| Sample 1 | |
| --- | --- |
| Variable | GOLD_stage |
| Filter | No Reintubation |
| Sample 2 | |
| Variable | GOLD_stage |
| Filter | Reintubation |

|  | Sample 1 | Sample 2 |
| --- | --- | --- |
| Sample size | 9 | 5 |
| Arithmetic mean | 2.8889 | 2.6000 |
| 95% CI for the mean | 2.2880 to 3.4898 | 1.4894 to 3.7106 |
| Variance | 0.6111 | 0.8000 |
| Standard deviation | 0.7817 | 0.8944 |
| Standard error of the mean | 0.2606 | 0.4000 |

| F-test for equal variances | P = 0.689 |
| --- | --- |

## T-test (assuming equal variances)

| Difference | -0.2889 |
| --- | --- |
| Standard Error | 0.4579 |
| 95% CI of difference | -1.2867 to 0.7089 |
| Test statistic t | -0.631 |
| Degrees of Freedom (DF) | 12 |
| Two-tailed probability | P = 0.5400 |

Independent samples t-test

| Sample 1 | |
| --- | --- |
| Variable | SAPS II (at ICU admission) |
| Filter | No Reintubation |
| Sample 2 | |
| Variable | SAPS II (at ICU admission) |
| Filter | Reintubation |

|  | Sample 1 | Sample 2 |
| --- | --- | --- |
| Sample size | 9 | 5 |
| Arithmetic mean | 41.1111 | 37.0000 |
| 95% CI for the mean | 28.6962 to 53.5260 | 29.9214 to 44.0786 |
| Variance | 260.8611 | 32.5000 |
| Standard deviation | 16.1512 | 5.7009 |
| Standard error of the mean | 5.3837 | 2.5495 |

| F-test for equal variances | P = 0.061 |
| --- | --- |

## T-test (assuming equal variances)

| Difference | -4.1111 |
| --- | --- |
| Standard Error | 7.5812 |
| 95% CI of difference | -20.6292 to 12.4069 |
| Test statistic t | -0.542 |
| Degrees of Freedom (DF) | 12 |
| Two-tailed probability | P = 0.5976 |

Independent samples t-test

| Sample 1 | |
| --- | --- |
| Variable | SOFA (day of study) |
| Filter | No Reintubation |
| Sample 2 | |
| Variable | SOFA (day of study) |
| Filter | Reintubation |

|  | Sample 1 | Sample 2 |
| --- | --- | --- |
| Sample size | 9 | 5 |
| Arithmetic mean | 6.2222 | 4.6000 |
| 95% CI for the mean | 3.9885 to 8.4559 | 3.1843 to 6.0157 |
| Variance | 8.4444 | 1.3000 |
| Standard deviation | 2.9059 | 1.1402 |
| Standard error of the mean | 0.9686 | 0.5099 |

| F-test for equal variances | P = 0.088 |
| --- | --- |

## T-test (assuming equal variances)

| Difference | -1.6222 |
| --- | --- |
| Standard Error | 1.3734 |
| 95% CI of difference | -4.6146 to 1.3702 |
| Test statistic t | -1.181 |
| Degrees of Freedom (DF) | 12 |
| Two-tailed probability | P = 0.2604 |
