# Supplementary material for: High-flow nasal cannula oxygen therapy decreases postextubation neuroventilatory drive and work of breathing in patients with chronic obstructive pulmonary disease
Source: Crit Care. 2018 Aug 2;22:180. doi: 10.1186/s13054-018-2107-9 (PMC6091018; doi:10.1186/s13054-018-2107-9)
Supplement: Supplementary file 2 — Independent sample t tests. Comparison between patients who required reintubation and patients who were successfully extubated in terms of EAdi parameters or work of breathing during each experimental condition. (DOCX 42 kb) [file 13054_2018_2107_MOESM2_ESM.docx]

Independent samples t-test

| Sample 1 | |
| --- | --- |
| Variable | Eadi_Peak |
| Select | Not Reintubated HFNC1 |
| Sample 2 | |
| Variable | Eadi_Peak |
| Select | Reintubated HFNC1 |

|  | Sample 1 | Sample 2 |
| --- | --- | --- |
| Sample size | 9 | 5 |
| Arithmetic mean | 15.7467 | 14.7725 |
| 95% CI for the mean | 10.9729 to 20.5205 | 5.4317 to 24.1133 |
| Variance | 38.5703 | 56.5930 |
| Standard deviation | 6.2105 | 7.5228 |
| Standard error of the mean | 2.0702 | 3.3643 |

| F-test for equal variances | P = 0.596 |
| --- | --- |

## T-test (assuming equal variances)

| Difference | -0.9742 |
| --- | --- |
| Standard Error | 3.7241 |
| 95% CI of difference | -9.0882 to 7.1399 |
| Test statistic t | -0.262 |
| Degrees of Freedom (DF) | 12 |
| Two-tailed probability | P = 0.7981 |

Independent samples t-test

| Sample 1 | |
| --- | --- |
| Variable | Slope |
| Select | Not Reintubated HFNC1 |
| Sample 2 | |
| Variable | Slope |
| Select | Reintubated HFNC1 |

|  | Sample 1 | Sample 2 |
| --- | --- | --- |
| Sample size | 9 | 5 |
| Arithmetic mean | 19.7246 | 16.7488 |
| 95% CI for the mean | 11.3642 to 28.0849 | 0.5262 to 32.9715 |
| Variance | 118.2966 | 170.7012 |
| Standard deviation | 10.8764 | 13.0653 |
| Standard error of the mean | 3.6255 | 5.8430 |

| F-test for equal variances | P = 0.609 |
| --- | --- |

## T-test (assuming equal variances)

| Difference | -2.9757 |
| --- | --- |
| Standard Error | 6.4991 |
| 95% CI of difference | -17.1360 to 11.1845 |
| Test statistic t | -0.458 |
| Degrees of Freedom (DF) | 12 |
| Two-tailed probability | P = 0.6552 |

Independent samples t-test

| Sample 1 | |
| --- | --- |
| Variable | PTP tot |
| Select | Not Reintubated HFNC1 |
| Sample 2 | |
| Variable | PTP tot |
| Select | Reintubated HFNC1 |

|  | Sample 1 | Sample 2 |
| --- | --- | --- |
| Sample size | 9 | 5 |
| Arithmetic mean | 15.5012 | 10.5193 |
| 95% CI for the mean | 10.4934 to 20.5091 | 3.2119 to 17.8268 |
| Variance | 42.4454 | 34.6355 |
| Standard deviation | 6.5150 | 5.8852 |
| Standard error of the mean | 2.1717 | 2.6319 |

| F-test for equal variances | P = 0.901 |
| --- | --- |

## T-test (assuming equal variances)

| Difference | -4.9819 |
| --- | --- |
| Standard Error | 3.5207 |
| 95% CI of difference | -12.6529 to 2.6890 |
| Test statistic t | -1.415 |
| Degrees of Freedom (DF) | 12 |
| Two-tailed probability | P = 0.1825 |

Independent samples t-test

| Sample 1 | |
| --- | --- |
| Variable | Slope |
| Select | Not Reintubated HFNC1 |
| Sample 2 | |
| Variable | Slope |
| Select | Reintubated HFNC1 |

|  | Sample 1 | Sample 2 |
| --- | --- | --- |
| Sample size | 9 | 5 |
| Arithmetic mean | 19.7246 | 16.7488 |
| 95% CI for the mean | 11.3642 to 28.0849 | 0.5262 to 32.9715 |
| Variance | 118.2966 | 170.7012 |
| Standard deviation | 10.8764 | 13.0653 |
| Standard error of the mean | 3.6255 | 5.8430 |

| F-test for equal variances | P = 0.609 |
| --- | --- |

## T-test (assuming equal variances)

| Difference | -2.9757 |
| --- | --- |
| Standard Error | 6.4991 |
| 95% CI of difference | -17.1360 to 11.1845 |
| Test statistic t | -0.458 |
| Degrees of Freedom (DF) | 12 |
| Two-tailed probability | P = 0.6552 |

Independent samples t-test

| Sample 1 | |
| --- | --- |
| Variable | T insp Neural |
| Select | Not Reintubated HFNC1 |
| Sample 2 | |
| Variable | T insp Neural |
| Select | Reintubated HFNC1 |

|  | Sample 1 | Sample 2 |
| --- | --- | --- |
| Sample size | 9 | 5 |
| Arithmetic mean | 0.9809 | 0.8085 |
| 95% CI for the mean | 0.8355 to 1.1263 | 0.5477 to 1.0692 |
| Variance | 0.03578 | 0.04410 |
| Standard deviation | 0.1891 | 0.2100 |
| Standard error of the mean | 0.06305 | 0.09392 |

| F-test for equal variances | P = 0.740 |
| --- | --- |

## T-test (assuming equal variances)

| Difference | -0.1724 |
| --- | --- |
| Standard Error | 0.1095 |
| 95% CI of difference | -0.4110 to 0.06619 |
| Test statistic t | -1.574 |
| Degrees of Freedom (DF) | 12 |
| Two-tailed probability | P = 0.1414 |

Independent samples t-test

| Sample 1 | |
| --- | --- |
| Variable | Eadi Peak |
| Select | Not Reintubated Conventional O2 mask |
| Sample 2 | |
| Variable | Eadi Peak |
| Select | Reintubated Conventional O2 mask |

|  | Sample 1 | Sample 2 |
| --- | --- | --- |
| Sample size | 9 | 5 |
| Arithmetic mean | 24.4921 | 22.0630 |
| 95% CI for the mean | 15.5092 to 33.4749 | 11.0766 to 33.0494 |
| Variance | 136.5685 | 78.2893 |
| Standard deviation | 11.6863 | 8.8481 |
| Standard error of the mean | 3.8954 | 3.9570 |

| F-test for equal variances | P = 0.620 |
| --- | --- |

## T-test (assuming equal variances)

| Difference | -2.4290 |
| --- | --- |
| Standard Error | 6.0369 |
| 95% CI of difference | -15.5823 to 10.7242 |
| Test statistic t | -0.402 |
| Degrees of Freedom (DF) | 12 |
| Two-tailed probability | P = 0.6945 |

Independent samples t-test

| Sample 1 | |
| --- | --- |
| Variable | PTP breath |
| Select | Not Reintubated Conventional O2 mask |
| Sample 2 | |
| Variable | PTP breath |
| Select | Reintubated Conventional O2 mask |

|  | Sample 1 | Sample 2 |
| --- | --- | --- |
| Sample size | 9 | 5 |
| Arithmetic mean | 10.9467 | 8.1878 |
| 95% CI for the mean | 8.4720 to 13.4214 | 5.4010 to 10.9745 |
| Variance | 10.3652 | 5.0371 |
| Standard deviation | 3.2195 | 2.2443 |
| Standard error of the mean | 1.0732 | 1.0037 |

| F-test for equal variances | P = 0.507 |
| --- | --- |

## T-test (assuming equal variances)

| Difference | -2.7590 |
| --- | --- |
| Standard Error | 1.6347 |
| 95% CI of difference | -6.3206 to 0.8027 |
| Test statistic t | -1.688 |
| Degrees of Freedom (DF) | 12 |
| Two-tailed probability | P = 0.1173 |

Independent samples t-test

| Sample 1 | |
| --- | --- |
| Variable | PTP tot |
| Select | Not Reintubated Conventional O2 mask |
| Sample 2 | |
| Variable | PTP tot |
| Select | Reintubated Conventional O2 mask |

|  | Sample 1 | Sample 2 |
| --- | --- | --- |
| Sample size | 9 | 5 |
| Arithmetic mean | 22.6434 | 18.1813 |
| 95% CI for the mean | 12.3944 to 32.8923 | 7.1348 to 29.2279 |
| Variance | 177.7792 | 79.1488 |
| Standard deviation | 13.3334 | 8.8966 |
| Standard error of the mean | 4.4445 | 3.9787 |

| F-test for equal variances | P = 0.453 |
| --- | --- |

## T-test (assuming equal variances)

| Difference | -4.4621 |
| --- | --- |
| Standard Error | 6.7142 |
| 95% CI of difference | -19.0911 to 10.1670 |
| Test statistic t | -0.665 |
| Degrees of Freedom (DF) | 12 |
| Two-tailed probability | P = 0.5189 |

Independent samples t-test

| Sample 1 | |
| --- | --- |
| Variable | Slope |
| Select | Not Reintubated Conventional O2 mask |
| Sample 2 | |
| Variable | Slope |
| Select | Reintubated Conventional O2 mask |

|  | Sample 1 | Sample 2 |
| --- | --- | --- |
| Sample size | 9 | 5 |
| Arithmetic mean | 25.1684 | 22.0057 |
| 95% CI for the mean | 13.5680 to 36.7689 | 2.8311 to 41.1803 |
| Variance | 227.7573 | 238.4754 |
| Standard deviation | 15.0916 | 15.4426 |
| Standard error of the mean | 5.0305 | 6.9062 |

| F-test for equal variances | P = 0.881 |
| --- | --- |

## T-test (assuming equal variances)

| Difference | -3.1627 |
| --- | --- |
| Standard Error | 8.4835 |
| 95% CI of difference | -21.6466 to 15.3212 |
| Test statistic t | -0.373 |
| Degrees of Freedom (DF) | 12 |
| Two-tailed probability | P = 0.7158 |

Independent samples t-test

| Sample 1 | |
| --- | --- |
| Variable | T_insp_Neural T insp Neural |
| Select | Not Reintubated Conventional O2 mask |
| Sample 2 | |
| Variable | T_insp_Neural T insp Neural |
| Select | Reintubated Conventional O2 mask |

|  | Sample 1 | Sample 2 |
| --- | --- | --- |
| Sample size | 9 | 5 |
| Arithmetic mean | 0.9419 | 0.9174 |
| 95% CI for the mean | 0.7734 to 1.1105 | 0.6674 to 1.1673 |
| Variance | 0.04810 | 0.04053 |
| Standard deviation | 0.2193 | 0.2013 |
| Standard error of the mean | 0.07311 | 0.09004 |

| F-test for equal variances | P = 0.929 |
| --- | --- |

## T-test (assuming equal variances)

| Difference | -0.02458 |
| --- | --- |
| Standard Error | 0.1191 |
| 95% CI of difference | -0.2840 to 0.2349 |
| Test statistic t | -0.206 |
| Degrees of Freedom (DF) | 12 |
| Two-tailed probability | P = 0.8399 |

Independent samples t-test

| Sample 1 | |
| --- | --- |
| Variable | Eadi Peak |
| Select | Not Reintubated HFNC2 |
| Sample 2 | |
| Variable | Eadi Peak |
| Select | Reintubated HFNC2 |

|  | Sample 1 | Sample 2 |
| --- | --- | --- |
| Sample size | 9 | 5 |
| Arithmetic mean | 15.3095 | 14.4903 |
| 95% CI for the mean | 10.6936 to 19.9254 | 4.7862 to 24.1944 |
| Variance | 36.0607 | 61.0804 |
| Standard deviation | 6.0051 | 7.8154 |
| Standard error of the mean | 2.0017 | 3.4952 |

| F-test for equal variances | P = 0.487 |
| --- | --- |

## T-test (assuming equal variances)

| Difference | -0.8192 |
| --- | --- |
| Standard Error | 3.7167 |
| 95% CI of difference | -8.9171 to 7.2787 |
| Test statistic t | -0.220 |
| Degrees of Freedom (DF) | 12 |
| Two-tailed probability | P = 0.8293 |

Independent samples t-test

| Sample 1 | |
| --- | --- |
| Variable | PTP breath |
| Select | Not Reintubated HFNC2 |
| Sample 2 | |
| Variable | PTP breath |
| Select | Reintubated HFNC2 |

|  | Sample 1 | Sample 2 |
| --- | --- | --- |
| Sample size | 9 | 5 |
| Arithmetic mean | 7.7269 | 4.8146 |
| 95% CI for the mean | 5.5785 to 9.8753 | 2.2452 to 7.3839 |
| Variance | 7.8119 | 4.2819 |
| Standard deviation | 2.7950 | 2.0693 |
| Standard error of the mean | 0.9317 | 0.9254 |

| F-test for equal variances | P = 0.588 |
| --- | --- |

## T-test (assuming equal variances)

| Difference | -2.9123 |
| --- | --- |
| Standard Error | 1.4368 |
| 95% CI of difference | -6.0427 to 0.2181 |
| Test statistic t | -2.027 |
| Degrees of Freedom (DF) | 12 |
| Two-tailed probability | P = 0.0655 |

Independent samples t-test

| Sample 1 | |
| --- | --- |
| Variable | PTP tot |
| Select | Not Reintubated HFNC2 |
| Sample 2 | |
| Variable | PTP tot |
| Select | Reintubated HFNC2 |

|  | Sample 1 | Sample 2 |
| --- | --- | --- |
| Sample size | 9 | 5 |
| Arithmetic mean | 12.9667 | 10.6528 |
| 95% CI for the mean | 9.4170 to 16.5163 | 2.7092 to 18.5964 |
| Variance | 21.3254 | 40.9287 |
| Standard deviation | 4.6179 | 6.3976 |
| Standard error of the mean | 1.5393 | 2.8611 |

| F-test for equal variances | P = 0.401 |
| --- | --- |

## T-test (assuming equal variances)

| Difference | -2.3139 |
| --- | --- |
| Standard Error | 2.9441 |
| 95% CI of difference | -8.7285 to 4.1006 |
| Test statistic t | -0.786 |
| Degrees of Freedom (DF) | 12 |
| Two-tailed probability | P = 0.4471 |

Independent samples t-test

| Sample 1 | |
| --- | --- |
| Variable | Slope |
| Select | Not Reintubated HFNC2 |
| Sample 2 | |
| Variable | Slope |
| Select | Reintubated HFNC2 |

|  | Sample 1 | Sample 2 |
| --- | --- | --- |
| Sample size | 9 | 5 |
| Arithmetic mean | 17.8504 | 17.0648 |
| 95% CI for the mean | 9.7471 to 25.9537 | 3.6589 to 30.4706 |
| Variance | 111.1339 | 116.5686 |
| Standard deviation | 10.5420 | 10.7967 |
| Standard error of the mean | 3.5140 | 4.8284 |

| F-test for equal variances | P = 0.880 |
| --- | --- |

## T-test (assuming equal variances)

| Difference | -0.7856 |
| --- | --- |
| Standard Error | 5.9278 |
| 95% CI of difference | -13.7012 to 12.1299 |
| Test statistic t | -0.133 |
| Degrees of Freedom (DF) | 12 |
| Two-tailed probability | P = 0.8968 |

Independent samples t-test

| Sample 1 | |
| --- | --- |
| Variable | T insp Neural |
| Select | Not Reintubated HFNC2 |
| Sample 2 | |
| Variable | T insp Neural |
| Select | Reintubated HFNC2 |

|  | Sample 1 | Sample 2 |
| --- | --- | --- |
| Sample size | 9 | 5 |
| Arithmetic mean | 0.9895 | 0.8042 |
| 95% CI for the mean | 0.8791 to 1.0999 | 0.5896 to 1.0189 |
| Variance | 0.02063 | 0.02989 |
| Standard deviation | 0.1436 | 0.1729 |
| Standard error of the mean | 0.04788 | 0.07731 |

| F-test for equal variances | P = 0.606 |
| --- | --- |

## T-test (assuming equal variances)

| Difference | -0.1853 |
| --- | --- |
| Standard Error | 0.08590 |
| 95% CI of difference | -0.3724 to 0.001886 |
| Test statistic t | -2.157 |
| Degrees of Freedom (DF) | 12 |
| Two-tailed probability | P = 0.0520 |
