# Supplementary material for: High-flow nasal cannula oxygen therapy decreases postextubation neuroventilatory drive and work of breathing in patients with chronic obstructive pulmonary disease
Source: Crit Care. 2018 Aug 2;22:180. doi: 10.1186/s13054-018-2107-9 (PMC6091018; doi:10.1186/s13054-018-2107-9)
Supplement: Supplementary file 3 — Independent sample t tests. Comparison between patients admitted for hypercapnic respiratory failure due to an exacerbation of COPD and patients with a background of COPD but whose hypercapnic respiratory failure was due to other precipitating causes in terms of EAdi parameters or work of breathing during each experimental condition. (DOCX 60 kb) [file 13054_2018_2107_MOESM3_ESM.docx]

**Independent samples t-test**

| Sample 1 | |
| --- | --- |
| Variable | Eadi Peak |
| Select | No COPD Exacerbation HFNC1 |
| Sample 2 | |
| Variable | Eadi Peak |
| Select | COPD Exacerbation HFNC1 |

|  | Sample 1 | Sample 2 |
| --- | --- | --- |
| Sample size | 6 | 8 |
| Arithmetic mean | 18.4564 | 13.1055 |
| 95% CI for the mean | 13.0178 to 23.8950 | 7.5823 to 18.6288 |
| Variance | 26.8575 | 43.6476 |
| Standard deviation | 5.1824 | 6.6066 |
| Standard error of the mean | 2.1157 | 2.3358 |

| F-test for equal variances | P = 0.613 |
| --- | --- |

**T-test (assuming equal variances)**

| Difference | -5.3508 |
| --- | --- |
| Standard Error | 3.2696 |
| 95% CI of difference | -12.4746 to 1.7729 |
| Test statistic t | -1.637 |
| Degrees of Freedom (DF) | 12 |
| Two-tailed probability | P = 0.1277 |

**Independent samples t-test**

| Sample 1 | |
| --- | --- |
| Variable | PTP tot |
| Select | No COPD Exacerbation HFNC1 |
| Sample 2 | |
| Variable | Eadi Peak |
| Select | COPD Exacerbation HFNC1 |

|  | Sample 1 | Sample 2 |
| --- | --- | --- |
| Sample size | 6 | 8 |
| Arithmetic mean | 16.3352 | 13.1055 |
| 95% CI for the mean | 9.5830 to 23.0874 | 7.5823 to 18.6288 |
| Variance | 41.3982 | 43.6476 |
| Standard deviation | 6.4341 | 6.6066 |
| Standard error of the mean | 2.6267 | 2.3358 |

| F-test for equal variances | P = 0.989 |
| --- | --- |

**T-test (assuming equal variances)**

| Difference | -3.2297 |
| --- | --- |
| Standard Error | 3.5295 |
| 95% CI of difference | -10.9197 to 4.4604 |
| Test statistic t | -0.915 |
| Degrees of Freedom (DF) | 12 |
| Two-tailed probability | P = 0.3782 |

**Independent samples t-test**

| Sample 1 | |
| --- | --- |
| Variable | T insp Neural |
| Select | No COPD Exacerbation HFNC1 |
| Sample 2 | |
| Variable | T insp Neural |
| Select | COPD Exacerbation HFNC1 |

|  | Sample 1 | Sample 2 |
| --- | --- | --- |
| Sample size | 6 | 8 |
| Arithmetic mean | 0.8576 | 0.9656 |
| 95% CI for the mean | 0.5969 to 1.1184 | 0.8210 to 1.1103 |
| Variance | 0.06173 | 0.02994 |
| Standard deviation | 0.2485 | 0.1730 |
| Standard error of the mean | 0.1014 | 0.06117 |

| F-test for equal variances | P = 0.372 |
| --- | --- |

**T-test (assuming equal variances)**

| Difference | 0.1080 |
| --- | --- |
| Standard Error | 0.1122 |
| 95% CI of difference | -0.1365 to 0.3525 |
| Test statistic t | 0.962 |
| Degrees of Freedom (DF) | 12 |
| Two-tailed probability | P = 0.3550 |

| [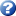](cmd:HELP) [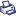](cmd:PRINT) | [Data comparison graph](cmd:DATACOMPPLOT) |
| --- | --- |

**Independent samples t-test**

| Sample 1 | |
| --- | --- |
| Variable | PO2 |
| Select | No COPD Exacerbation HFNC1 |
| Sample 2 | |
| Variable | PO2 |
| Select | COPD Exacerbation HFNC1 |

|  | Sample 1 | Sample 2 |
| --- | --- | --- |
| Sample size | 6 | 8 |
| Arithmetic mean | 76.8333 | 74.5250 |
| 95% CI for the mean | 69.3120 to 84.3547 | 68.6666 to 80.3834 |
| Variance | 51.3667 | 49.1050 |
| Standard deviation | 7.1671 | 7.0075 |
| Standard error of the mean | 2.9259 | 2.4775 |

| F-test for equal variances | P = 0.921 |
| --- | --- |

**T-test (assuming equal variances)**

| Difference | -2.3083 |
| --- | --- |
| Standard Error | 3.8206 |
| 95% CI of difference | -10.6328 to 6.0161 |
| Test statistic t | -0.604 |
| Degrees of Freedom (DF) | 12 |
| Two-tailed probability | P = 0.5570 |

| [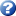](cmd:HELP) [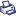](cmd:PRINT) | [Data comparison graph](cmd:DATACOMPPLOT) |
| --- | --- |

**Independent samples t-test**

| Sample 1 | |
| --- | --- |
| Variable | PCO2 |
| Select | No COPD Exacerbation HFNC1 |
| Sample 2 | |
| Variable | PCO2 |
| Select | COPD Exacerbation HFNC1 |

|  | Sample 1 | Sample 2 |
| --- | --- | --- |
| Sample size | 6 | 8 |
| Arithmetic mean | 49.2000 | 50.4125 |
| 95% CI for the mean | 31.2525 to 67.1475 | 44.2172 to 56.6078 |
| Variance | 292.4800 | 54.9155 |
| Standard deviation | 17.1020 | 7.4105 |
| Standard error of the mean | 6.9819 | 2.6200 |

| F-test for equal variances | P = 0.049 |
| --- | --- |

**Welch-test (assuming unequal variances)**

| Difference | 1.2125 |
| --- | --- |
| Standard Error | 7.4573 |
| 95% CI of difference | -17.0348 to 19.4598 |
| Test statistic t(d) | 0.163 |
| Degrees of Freedom (DF) | 6.4 |
| Two-tailed probability | P = 0.8762 |

| [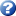](cmd:HELP) [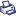](cmd:PRINT) | [Data comparison graph](cmd:DATACOMPPLOT) |
| --- | --- |

**Independent samples t-test**

| Sample 1 | |
| --- | --- |
| Variable | RR |
| Select | No COPD Exacerbation HFNC1 |
| Sample 2 | |
| Variable | RR |
| Select | COPD Exacerbation HFNC1 |

|  | Sample 1 | Sample 2 |
| --- | --- | --- |
| Sample size | 6 | 8 |
| Arithmetic mean | 20.8165 | 20.3543 |
| 95% CI for the mean | 18.0208 to 23.6121 | 17.6115 to 23.0971 |
| Variance | 7.0966 | 10.7632 |
| Standard deviation | 2.6639 | 3.2807 |
| Standard error of the mean | 1.0875 | 1.1599 |

| F-test for equal variances | P = 0.668 |
| --- | --- |

**T-test (assuming equal variances)**

| Difference | -0.4622 |
| --- | --- |
| Standard Error | 1.6412 |
| 95% CI of difference | -4.0381 to 3.1138 |
| Test statistic t | -0.282 |
| Degrees of Freedom (DF) | 12 |
| Two-tailed probability | P = 0.7831 |

| [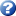](cmd:HELP) [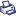](cmd:PRINT) | [Data comparison graph](cmd:DATACOMPPLOT) |
| --- | --- |
|  |  |

**Independent samples t-test**

| Sample 1 | |
| --- | --- |
| Variable | Eadi Peak |
| Select | Not COPD Exacerbation Conventional O2 mask |
| Sample 2 | |
| Variable | Eadi Peak |
| Select | COPD Exacerbation Conventional O2 mask |

|  | Sample 1 | Sample 2 |
| --- | --- | --- |
| Sample size | 6 | 8 |
| Arithmetic mean | 29.0209 | 19.5773 |
| 95% CI for the mean | 20.0498 to 37.9920 | 10.9034 to 28.2512 |
| Variance | 73.0771 | 107.6457 |
| Standard deviation | 8.5485 | 10.3752 |
| Standard error of the mean | 3.4899 | 3.6682 |

| F-test for equal variances | P = 0.692 |
| --- | --- |

**T-test (assuming equal variances)**

| Difference | -9.4436 |
| --- | --- |
| Standard Error | 5.2149 |
| 95% CI of difference | -20.8060 to 1.9188 |
| Test statistic t | -1.811 |
| Degrees of Freedom (DF) | 12 |
| Two-tailed probability | P = 0.0953 |

| [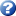](cmd:HELP) [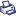](cmd:PRINT) | [Data comparison graph](cmd:DATACOMPPLOT) |
| --- | --- |

**Independent samples t-test**

| Sample 1 | |
| --- | --- |
| Variable | PTP_tot PTP tot |
| Select | Not COPD Exacerbation Conventional O2 mask |
| Sample 2 | |
| Variable | PTP_tot PTP tot |
| Select | COPD Exacerbation Conventional O2 mask |

|  | Sample 1 | Sample 2 |
| --- | --- | --- |
| Sample size | 6 | 8 |
| Arithmetic mean | 26.5674 | 16.9116 |
| 95% CI for the mean | 13.0165 to 40.1182 | 8.8586 to 24.9646 |
| Variance | 166.7335 | 92.7860 |
| Standard deviation | 12.9125 | 9.6325 |
| Standard error of the mean | 5.2715 | 3.4056 |

| F-test for equal variances | P = 0.464 |
| --- | --- |

**T-test (assuming equal variances)**

| Difference | -9.6557 |
| --- | --- |
| Standard Error | 6.0041 |
| 95% CI of difference | -22.7375 to 3.4261 |
| Test statistic t | -1.608 |
| Degrees of Freedom (DF) | 12 |
| Two-tailed probability | P = 0.1338 |

| [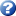](cmd:HELP) [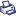](cmd:PRINT) | [Data comparison graph](cmd:DATACOMPPLOT) |
| --- | --- |

**Independent samples t-test**

| Sample 1 | |
| --- | --- |
| Variable | T_insp_Neural T insp Neural |
| Select | Not COPD Exacerbation Conventional O2 mask |
| Sample 2 | |
| Variable | T_insp_Neural T insp Neural |
| Select | COPD Exacerbation Conventional O2 mask |

|  | Sample 1 | Sample 2 |
| --- | --- | --- |
| Sample size | 6 | 8 |
| Arithmetic mean | 0.8980 | 0.9596 |
| 95% CI for the mean | 0.6513 to 1.1447 | 0.7986 to 1.1205 |
| Variance | 0.05527 | 0.03708 |
| Standard deviation | 0.2351 | 0.1926 |
| Standard error of the mean | 0.09597 | 0.06808 |

| F-test for equal variances | P = 0.608 |
| --- | --- |

**T-test (assuming equal variances)**

| Difference | 0.06158 |
| --- | --- |
| Standard Error | 0.1141 |
| 95% CI of difference | -0.1871 to 0.3102 |
| Test statistic t | 0.540 |
| Degrees of Freedom (DF) | 12 |
| Two-tailed probability | P = 0.5994 |

| [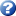](cmd:HELP) [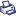](cmd:PRINT) | [Data comparison graph](cmd:DATACOMPPLOT) |
| --- | --- |

**Independent samples t-test**

| Sample 1 | |
| --- | --- |
| Variable | PO2 |
| Select | Not COPD Exacerbation Conventional O2 mask |
| Sample 2 | |
| Variable | PO2 |
| Select | COPD Exacerbation Conventional O2 mask |

|  | Sample 1 | Sample 2 |
| --- | --- | --- |
| Sample size | 6 | 8 |
| Arithmetic mean | 75.2833 | 71.0750 |
| 95% CI for the mean | 66.0721 to 84.4946 | 63.8571 to 78.2929 |
| Variance | 77.0417 | 74.5393 |
| Standard deviation | 8.7773 | 8.6336 |
| Standard error of the mean | 3.5833 | 3.0524 |

| F-test for equal variances | P = 0.932 |
| --- | --- |

**T-test (assuming equal variances)**

| Difference | -4.2083 |
| --- | --- |
| Standard Error | 4.6952 |
| 95% CI of difference | -14.4383 to 6.0216 |
| Test statistic t | -0.896 |
| Degrees of Freedom (DF) | 12 |
| Two-tailed probability | P = 0.3877 |

| [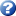](cmd:HELP) [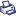](cmd:PRINT) | [Data comparison graph](cmd:DATACOMPPLOT) |
| --- | --- |

**Independent samples t-test**

| Sample 1 | |
| --- | --- |
| Variable | PCO2 |
| Select | Not COPD Exacerbation Conventional O2 mask |
| Sample 2 | |
| Variable | PCO2 |
| Select | COPD Exacerbation Conventional O2 mask |

|  | Sample 1 | Sample 2 |
| --- | --- | --- |
| Sample size | 6 | 8 |
| Arithmetic mean | 52.7667 | 51.1125 |
| 95% CI for the mean | 34.0906 to 71.4427 | 43.9353 to 58.2897 |
| Variance | 316.7067 | 73.7013 |
| Standard deviation | 17.7963 | 8.5849 |
| Standard error of the mean | 7.2653 | 3.0352 |

| F-test for equal variances | P = 0.083 |
| --- | --- |

**T-test (assuming equal variances)**

| Difference | -1.6542 |
| --- | --- |
| Standard Error | 7.1434 |
| 95% CI of difference | -17.2183 to 13.9100 |
| Test statistic t | -0.232 |
| Degrees of Freedom (DF) | 12 |
| Two-tailed probability | P = 0.8208 |

| [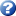](cmd:HELP) [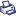](cmd:PRINT) | [Data comparison graph](cmd:DATACOMPPLOT) |
| --- | --- |

**Independent samples t-test**

| Sample 1 | |
| --- | --- |
| Variable | RR |
| Select | Not COPD Exacerbation Conventional O2 mask |
| Sample 2 | |
| Variable | RR |
| Select | COPD Exacerbation Conventional O2 mask |

|  | Sample 1 | Sample 2 |
| --- | --- | --- |
| Sample size | 6 | 8 |
| Arithmetic mean | 22.2343 | 20.8451 |
| 95% CI for the mean | 17.2079 to 27.2606 | 17.8327 to 23.8576 |
| Variance | 22.9400 | 12.9836 |
| Standard deviation | 4.7896 | 3.6033 |
| Standard error of the mean | 1.9553 | 1.2740 |

| F-test for equal variances | P = 0.476 |
| --- | --- |

**T-test (assuming equal variances)**

| Difference | -1.3891 |
| --- | --- |
| Standard Error | 2.2354 |
| 95% CI of difference | -6.2596 to 3.4813 |
| Test statistic t | -0.621 |
| Degrees of Freedom (DF) | 12 |
| Two-tailed probability | P = 0.5459 |

| [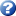](cmd:HELP) [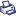](cmd:PRINT) | [Data comparison graph](cmd:DATACOMPPLOT) |
| --- | --- |

**Independent samples t-test**

| Sample 1 | |
| --- | --- |
| Variable | Eadi Peak |
| Select | Not COPD Exacerbation HFNC2 |
| Sample 2 | |
| Variable | Eadi Peak |
| Select | COPD Exacerbation HFNC2 |

|  | Sample 1 | Sample 2 |
| --- | --- | --- |
| Sample size | 6 | 8 |
| Arithmetic mean | 18.1204 | 12.6894 |
| 95% CI for the mean | 12.2846 to 23.9562 | 7.4093 to 17.9694 |
| Variance | 30.9234 | 39.8882 |
| Standard deviation | 5.5609 | 6.3157 |
| Standard error of the mean | 2.2702 | 2.2329 |

| F-test for equal variances | P = 0.806 |
| --- | --- |

**T-test (assuming equal variances)**

| Difference | -5.4310 |
| --- | --- |
| Standard Error | 3.2472 |
| 95% CI of difference | -12.5062 to 1.6441 |
| Test statistic t | -1.673 |
| Degrees of Freedom (DF) | 12 |
| Two-tailed probability | P = 0.1203 |

| [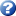](cmd:HELP) [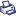](cmd:PRINT) | [Data comparison graph](cmd:DATACOMPPLOT) |
| --- | --- |

**Independent samples t-test**

| Sample 1 | |
| --- | --- |
| Variable | PTP_tot PTP tot |
| Select | Not COPD Exacerbation HFNC2 |
| Sample 2 | |
| Variable | PTP_tot PTP tot |
| Select | COPD Exacerbation HFNC2 |

|  | Sample 1 | Sample 2 |
| --- | --- | --- |
| Sample size | 6 | 8 |
| Arithmetic mean | 13.2649 | 11.2968 |
| 95% CI for the mean | 8.8940 to 17.6358 | 6.2856 to 16.3081 |
| Variance | 17.3475 | 35.9300 |
| Standard deviation | 4.1650 | 5.9942 |
| Standard error of the mean | 1.7004 | 2.1193 |

| F-test for equal variances | P = 0.440 |
| --- | --- |

**T-test (assuming equal variances)**

| Difference | -1.9681 |
| --- | --- |
| Standard Error | 2.8673 |
| 95% CI of difference | -8.2154 to 4.2792 |
| Test statistic t | -0.686 |
| Degrees of Freedom (DF) | 12 |
| Two-tailed probability | P = 0.5055 |

| [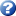](cmd:HELP) [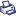](cmd:PRINT) | [Data comparison graph](cmd:DATACOMPPLOT) |
| --- | --- |

**Independent samples t-test**

| Sample 1 | |
| --- | --- |
| Variable | T insp Neural |
| Select | Not COPD Exacerbation HFNC2 |
| Sample 2 | |
| Variable | T insp Neural |
| Select | COPD Exacerbation HFNC2 |

|  | Sample 1 | Sample 2 |
| --- | --- | --- |
| Sample size | 6 | 8 |
| Arithmetic mean | 0.8884 | 0.9496 |
| 95% CI for the mean | 0.6791 to 1.0977 | 0.8143 to 1.0848 |
| Variance | 0.03977 | 0.02617 |
| Standard deviation | 0.1994 | 0.1618 |
| Standard error of the mean | 0.08142 | 0.05720 |

| F-test for equal variances | P = 0.592 |
| --- | --- |

**T-test (assuming equal variances)**

| Difference | 0.06120 |
| --- | --- |
| Standard Error | 0.09637 |
| 95% CI of difference | -0.1488 to 0.2712 |
| Test statistic t | 0.635 |
| Degrees of Freedom (DF) | 12 |
| Two-tailed probability | P = 0.5373 |

| [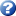](cmd:HELP) [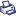](cmd:PRINT) | [Data comparison graph](cmd:DATACOMPPLOT) |
| --- | --- |

**Independent samples t-test**

| Sample 1 | |
| --- | --- |
| Variable | PO2 |
| Select | Not COPD Exacerbation HFNC2 |
| Sample 2 | |
| Variable | PO2 |
| Select | COPD Exacerbation HFNC2 |

|  | Sample 1 | Sample 2 |
| --- | --- | --- |
| Sample size | 6 | 8 |
| Arithmetic mean | 80.1667 | 82.0375 |
| 95% CI for the mean | 70.7004 to 89.6329 | 75.7839 to 88.2911 |
| Variance | 81.3667 | 55.9541 |
| Standard deviation | 9.0203 | 7.4802 |
| Standard error of the mean | 3.6825 | 2.6447 |

| F-test for equal variances | P = 0.629 |
| --- | --- |

**T-test (assuming equal variances)**

| Difference | 1.8708 |
| --- | --- |
| Standard Error | 4.4055 |
| 95% CI of difference | -7.7279 to 11.4696 |
| Test statistic t | 0.425 |
| Degrees of Freedom (DF) | 12 |
| Two-tailed probability | P = 0.6786 |

| [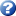](cmd:HELP) [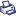](cmd:PRINT) | [Data comparison graph](cmd:DATACOMPPLOT) |
| --- | --- |

**Independent samples t-test**

| Sample 1 | |
| --- | --- |
| Variable | PCO2 |
| Select | Not COPD Exacerbation HFNC2 |
| Sample 2 | |
| Variable | PCO2 |
| Select | COPD Exacerbation HFNC2 |

|  | Sample 1 | Sample 2 |
| --- | --- | --- |
| Sample size | 6 | 8 |
| Arithmetic mean | 49.6833 | 50.4875 |
| 95% CI for the mean | 30.8577 to 68.5090 | 43.7216 to 57.2534 |
| Variance | 321.8017 | 65.4955 |
| Standard deviation | 17.9388 | 8.0929 |
| Standard error of the mean | 7.3235 | 2.8613 |

| F-test for equal variances | P = 0.060 |
| --- | --- |

**T-test (assuming equal variances)**

| Difference | 0.8042 |
| --- | --- |
| Standard Error | 7.0888 |
| 95% CI of difference | -14.6410 to 16.2493 |
| Test statistic t | 0.113 |
| Degrees of Freedom (DF) | 12 |
| Two-tailed probability | P = 0.9116 |

| [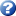](cmd:HELP) [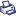](cmd:PRINT) | [Data comparison graph](cmd:DATACOMPPLOT) |
| --- | --- |

**Independent samples t-test**

| Sample 1 | |
| --- | --- |
| Variable | RR |
| Select | Not COPD Exacerbation HFNC2 |
| Sample 2 | |
| Variable | RR |
| Select | COPD Exacerbation HFNC2 |

|  | Sample 1 | Sample 2 |
| --- | --- | --- |
| Sample size | 6 | 8 |
| Arithmetic mean | 20.1612 | 19.9352 |
| 95% CI for the mean | 18.3228 to 21.9996 | 18.1943 to 21.6761 |
| Variance | 3.0689 | 4.3362 |
| Standard deviation | 1.7518 | 2.0823 |
| Standard error of the mean | 0.7152 | 0.7362 |

| F-test for equal variances | P = 0.727 |
| --- | --- |

**T-test (assuming equal variances)**

| Difference | -0.2260 |
| --- | --- |
| Standard Error | 1.0539 |
| 95% CI of difference | -2.5223 to 2.0702 |
| Test statistic t | -0.214 |
| Degrees of Freedom (DF) | 12 |
| Two-tailed probability | P = 0.8338 |

| [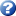](cmd:HELP) [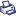](cmd:PRINT) | [Data comparison graph](cmd:DATACOMPPLOT) |
| --- | --- |
